# Supplementary material for: Mitochondrial dynamics controls anti-tumour innate immunity by regulating CHIP-IRF1 axis stability
Source: Nat Commun. 2017 Nov 27;8:1805. doi: 10.1038/s41467-017-01919-0 (PMC5703766; doi:10.1038/s41467-017-01919-0)
Supplement: Supplementary file 1 — Supplementary Information [file 41467_2017_1919_MOESM1_ESM.pdf]

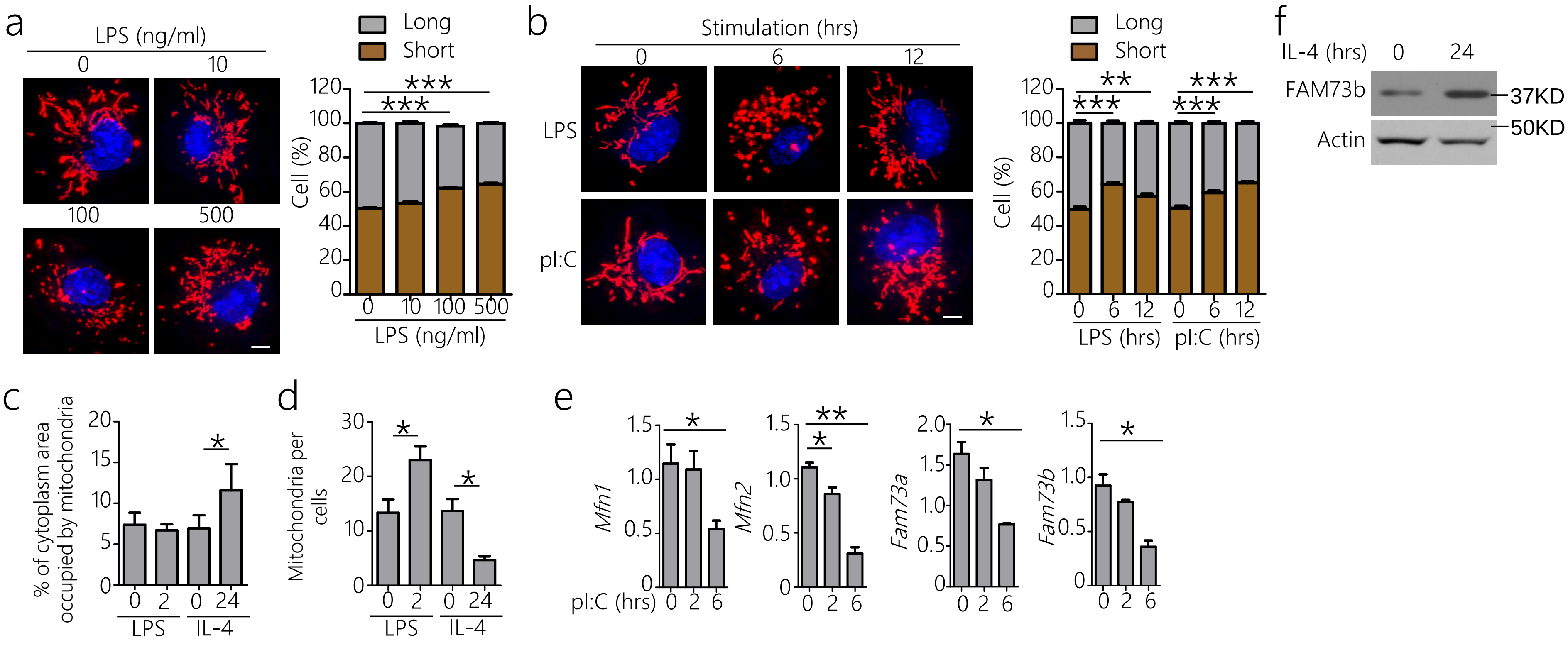

Supplementary Figure 1 Mitochondrial fission is drived by TLR stimulations (**a-b**) WT BMDMs treated with different doses of LPS (**a**) or time points (**b**) as indication time. Mitochondria were visualized using Mitospy^{TM} staining. (**c**) Cytoplasmic area occupied by mitochondria and mitochondria number (**d**) based on 5 replicate EM images. (**e**) qRT-PCR analyses of the indicated genes using poly I:C-stimulated BMDMs from WT mice. (**f**) IB analyses of Fam73b induction under IL-4 treatment. qPCR Data were normalized to a reference gene, *Actb*. Data shown are representative of three independent experiments. Scale bar in **a** and **b** are 5μm. Error bars are mean±SEM values. Two-tailed unpaired t-tests were performed and *P < 0.05; **P < 0.01; ***P<<0.005.

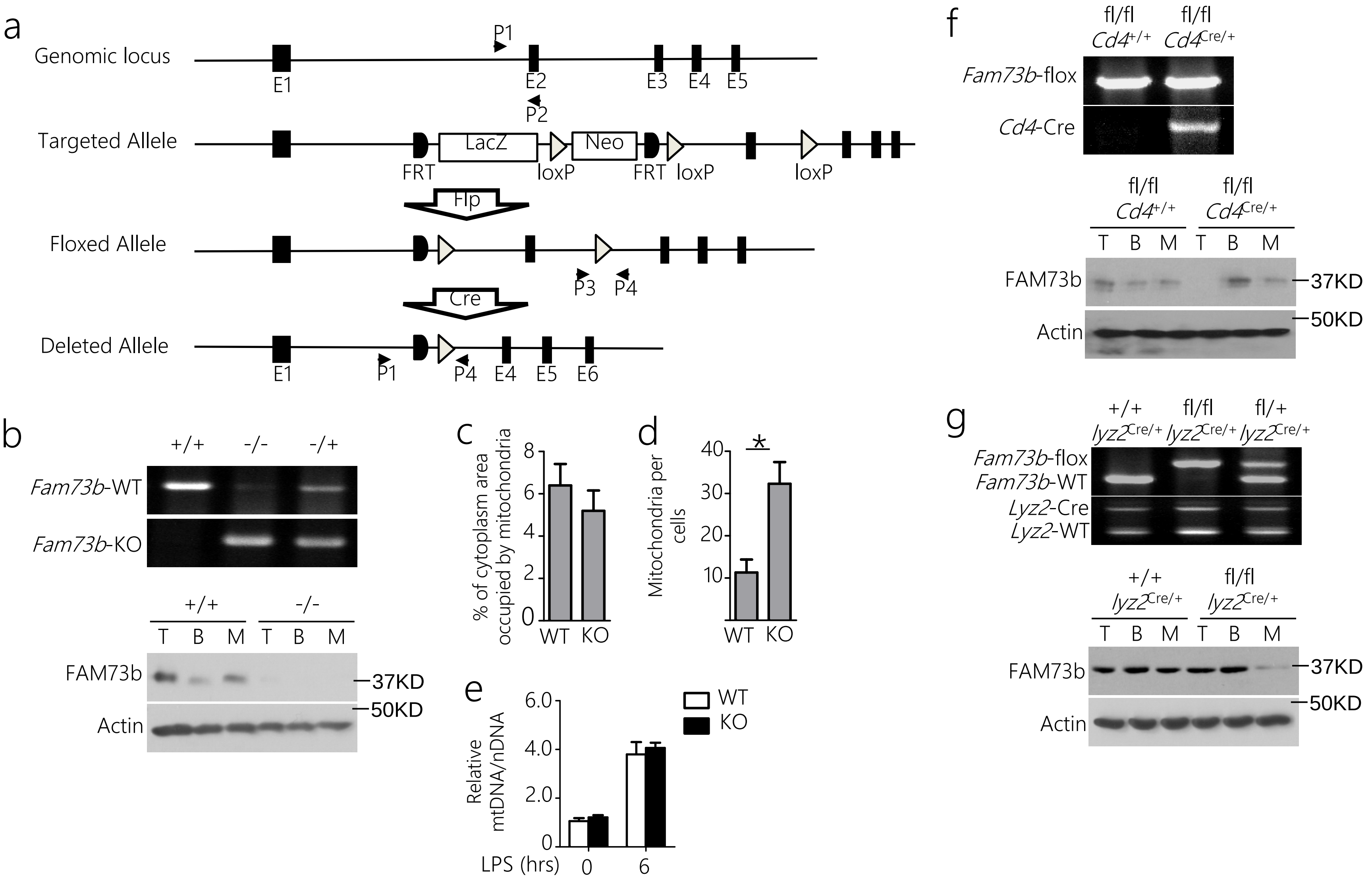

Supplementary Figure 2 ***Fam73b*** **gene targeting** (**a**) Schematic picture of *Fam73b* gene targeting using an FRT-LoxP vector, showing the the 5 exons of *Fam73b* gene. Targeted mice were crossed with FRT deleter (Rosa26-FLPe) mice to generate *Fam73b*-floxed mice, which were further crossed with different Cre mice to generate conditional KO mice. (**b**) Genotyping PCR of the germline KO mice. (**c-d**) The comparison of cytoplasmic area occupied by mitochondria and mitochondria number between WT and KO BMDMs . (**e**) mtDNA content normalized to nDNA in KO group compared to WT cells. (**f-g**) T cell-conditional KO (*Cd4*-Cre) (**f**) and myeloid-conditional KO (*Lyz2*-Cre) (**g**) mice using P3/P4 primer pair to amplify WT and floxed alleles (with the indicated sizes) and Cre-specific primers for the *Cd4*-Cre and *Lyz2*-Cre. IB was performed to measure the Fam73b protein level in the indicated cell types. All Data are representative of three independent experiments. Two-tailed unpaired t-tests were performed and *P < 0.05.

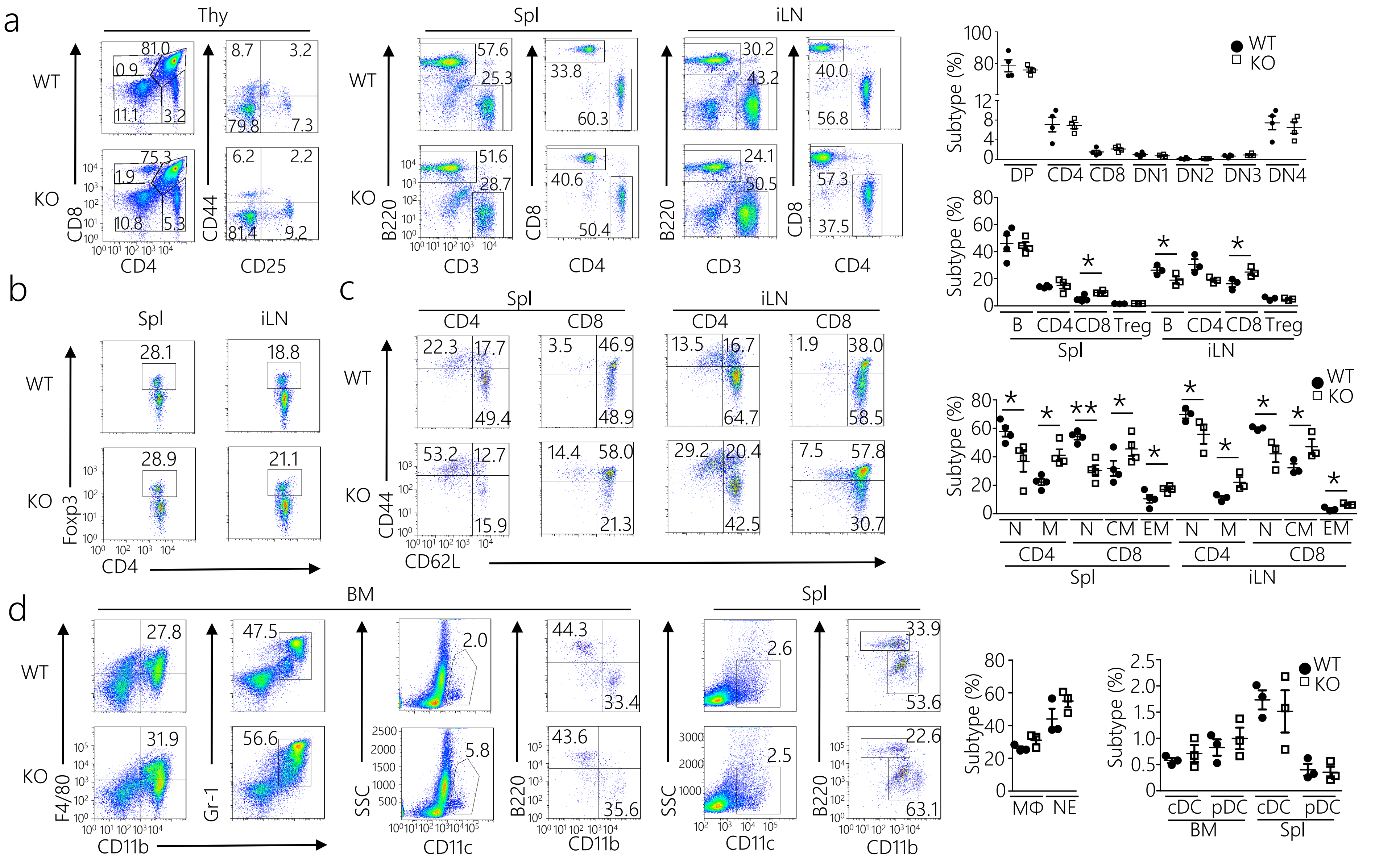

Supplementary Figure 3 Immune cell development in FAM73b KO mice Flow cytometry analysis of the percentage (numbers in quadrants) of different immune cells in the thymus (Thy), spleen (Spl) and inguinal lymph node (iLN) (**a**), Treg cells (**b**), naïve and memory T cells (**c**) of WT or FAM73b germline KO mice. (**d**) Flow cytometry analysis of macrophages ($CD11b^+F4/80^+$), Neutrophil ($CD11b^+GR\text{-}1^+$), conventional DC (cDC, $CD11c^+CD11b^{hi}B220^-$) and plasmacytoid DCs (pDCs, $CD11c^+CD11b^-B220^+$) in Bone marrow (BM) and Spleen (Spl). Data all panels are presented as representative FACS plots (left) and mean ± SEM values based on multiple samples (right). Similar results were obtained in three independent experiments. Two-tailed unpaired t-tests were performed and $^*P<0.05$; $^{**}P < 0.01$.

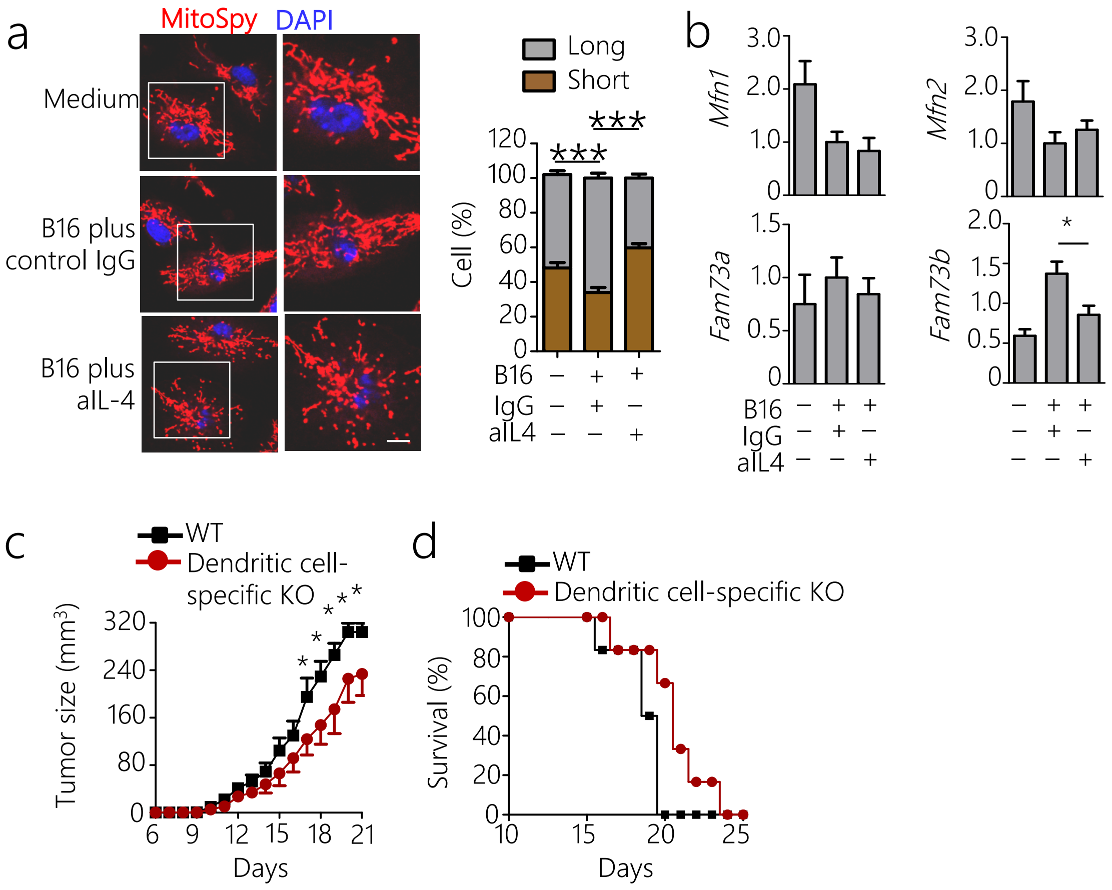

Supplementary Figure 4 Mitochondrial Fusion is promotes by tumor microenvironment (**a**) WT BMDMs co-cultured with B16 melanma cells with or without control IgG or αIL-4 (10mg/ml). Representative confocal images of the mitochondrial morphology were shown and quantification was analyzed with Image-Pro and performed as bar graph (right panel). (**b**) qRT-PCR analysis of the indicated genes using WT BMDMs treated as describing in **a**. (**c-d**) Tumor growth curve and survival curve of dendritic cell-specific KO mice (n=7) injected with 2 x 10^5 B16 melanoma cells. qPCR Data were normalized to a reference gene, *Actb*. Data shown are representative of three independent experiments. Scale bar in **a** is 5μm. Error bars are mean ± SEM values. Two-tailed unpaired t-tests were performed and *P < 0.05; **P < 0.01.

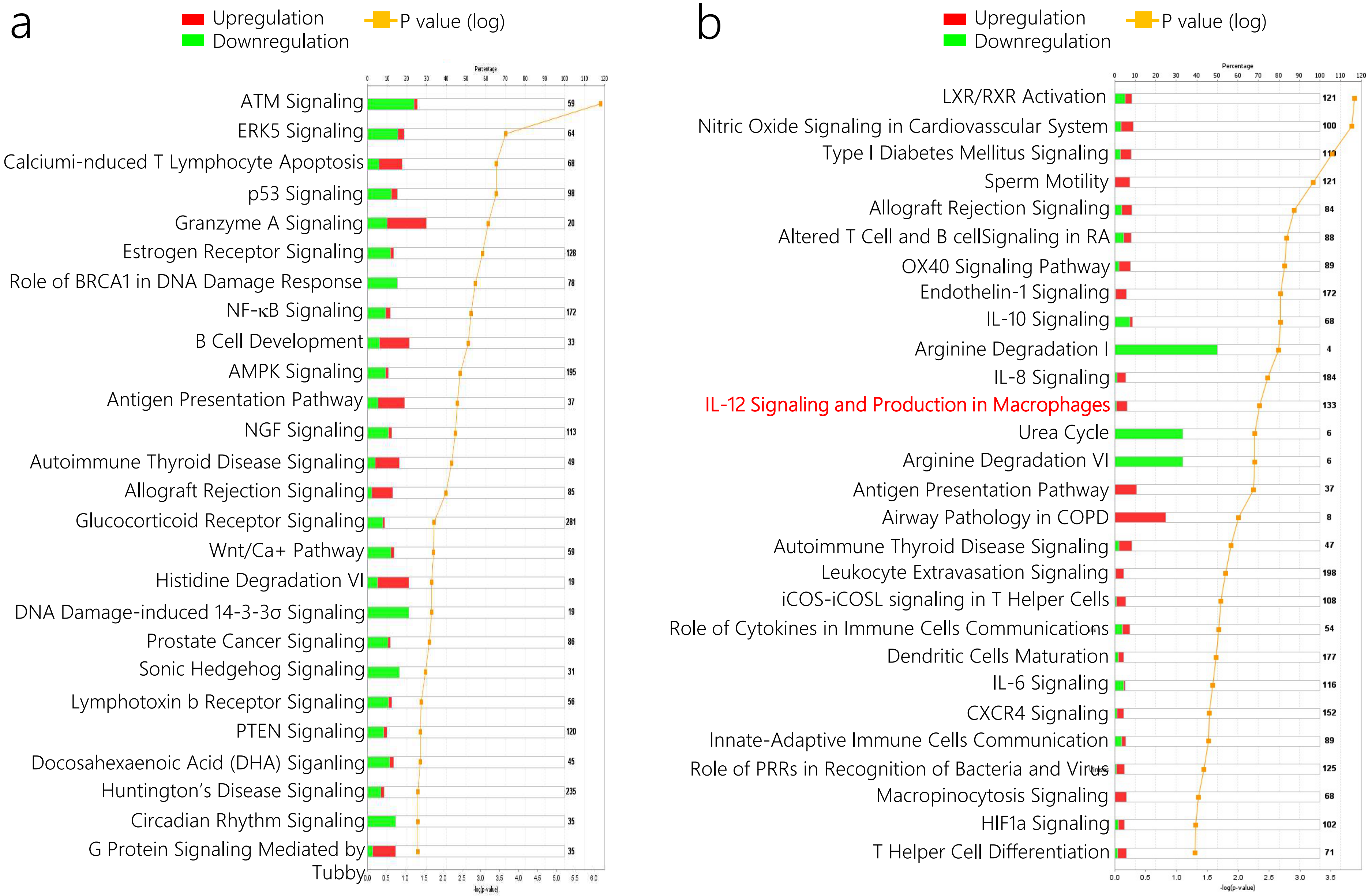

Supplementary Figure 5 Gene classification using Ingenuity Pathway Analysis (IPA) Green bars denote the percentage of downregulated and red bars the percentage of upregulated differentially expressed genes (DEG) relative to WT BMDMs without (**a**) or with (**b**) LPS stimulation in each pathway. Actual numbers of down- and upregulated DEG are shown inside bars. The yellow line denotes the likelihood [–log (P value)] that the specific pathway was affected by *Fam73b*.

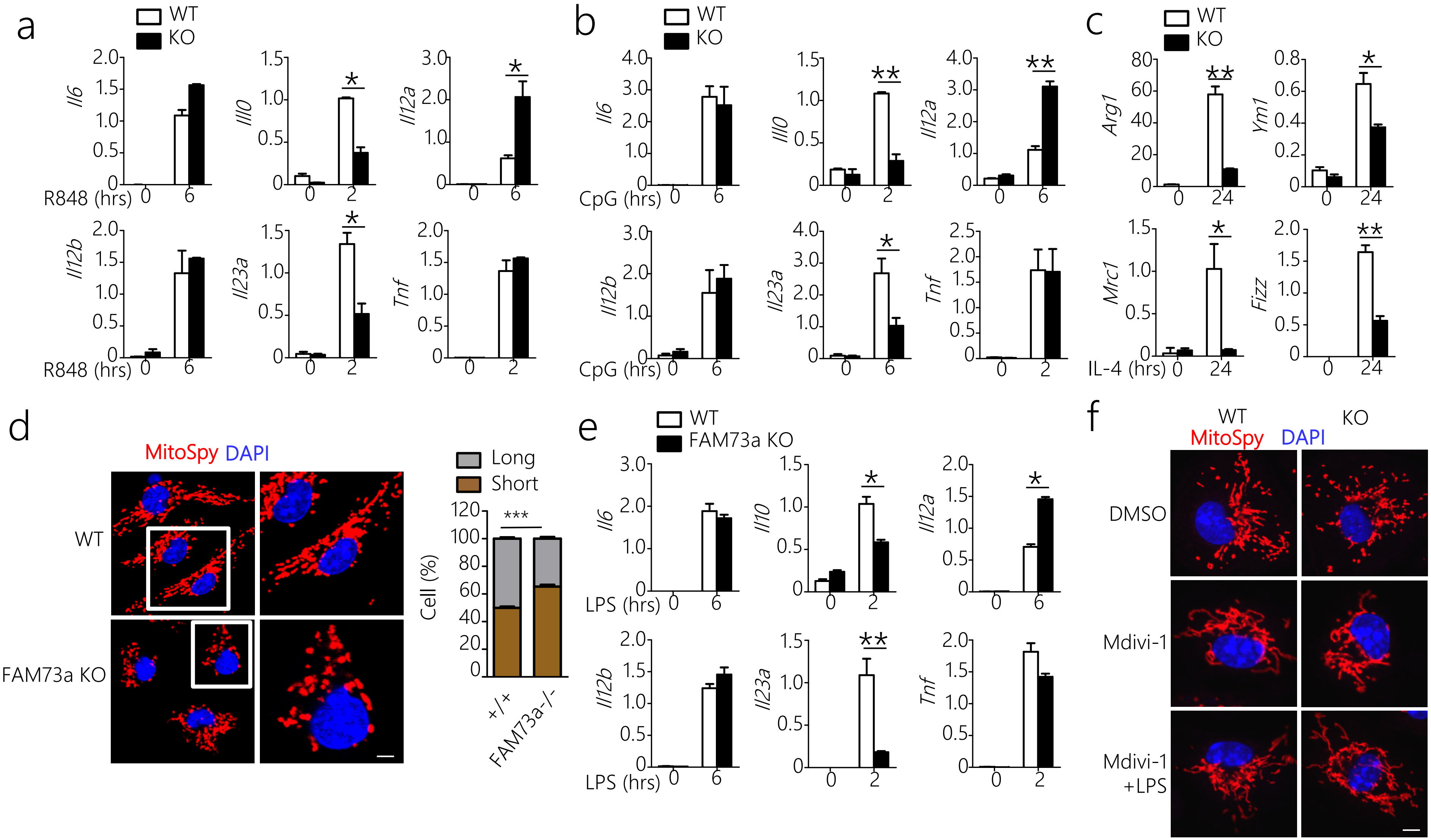

Supplementary Figure 6 Both FAM73a and FAM73b regulates TLR-induced inflammatory cytokiones qRT-PCR analysis of the indicated genes using WT or FAM73b KO BMDMs stimulated with R848 (**a**), CpG (**b**) and IL-4 (**c**). WT and FAM73a KO Mitochondria were visualized using MitospyTM orange CMTMRos staining. Representative confocal images and statics of the mitochondrial morphology were performed in (**d**) respectively. (**e**) qRT-PCR analysis of the indicated genes using WT or FAM73a KO BMDMs. (**f**) The mitochondrial morphology regulated by Mdivi-1 plus LPS was analyzed by confocal images . All qRT-PCR data are presented as fold relative to the *Actb* mRNA level. Similar results were obtained in three independent experiments. Scale bar in **d** and **f** are 5μm. Two-tailed unpaired t-tests were performed and $*P<0.05$; $**P < 0.01$.

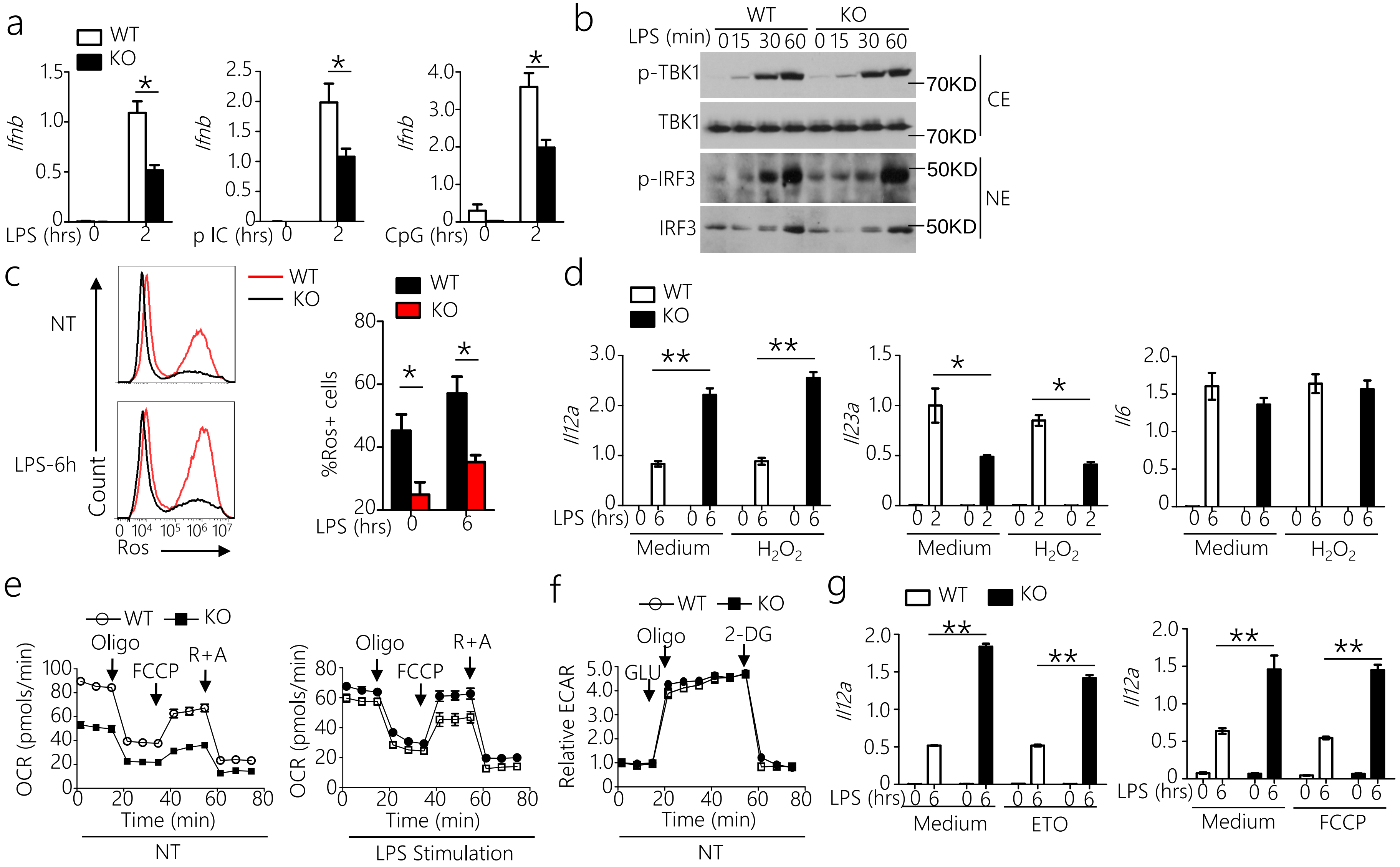

Supplementary Figure 7 Mitochondrial fission did not affect Type I IFNs or metabolic shift. (**a**) qRT-PCR analysis of *Ifnb* using WT or FAM73b KO BMDMs stimulated with stimulators. (**b**) IB analysis of the indicated protein levels in total-cell extracts of LPS-stimulated WT or FAM73b KO BMDMs. (**c**) LPS-stumlated WT and FAM73b KO BMDMs were stained with DCFH-DA, and subjected to flow cytometric analysis. The percentage of Ros positive cells was performed as bar graph. (**d**) WT and FAM73b KO BMDMs were incubated with 500 μM H_2O_2 for 2hrs, and qRT-PCR analysis of the indicated genes after LPS stimulation. (**e**) Mitochondrial fitness tests were used to compare OCR of WT and FAM73b KO BMDMs with or without LPS stimulation. (**f**) % ECAR of cells as **e** measured at baseline and after stimulation. (**g**) qRT-PCR analysis *Il12a* from WT or FAM73b KO BMDMs pretreated with ETO (**left panel**) or FCCP (**right panel**) plus LPS stimulation. All qRT-PCR data are presented as fold relative to the *Actb* mRNA level. Similar results were obtained in three independent experiments. Data represent mean ± SEM. Two-tailed unpaired t-tests were performed and $^{*}P<0.05$; $^{**}P < 0.01$.

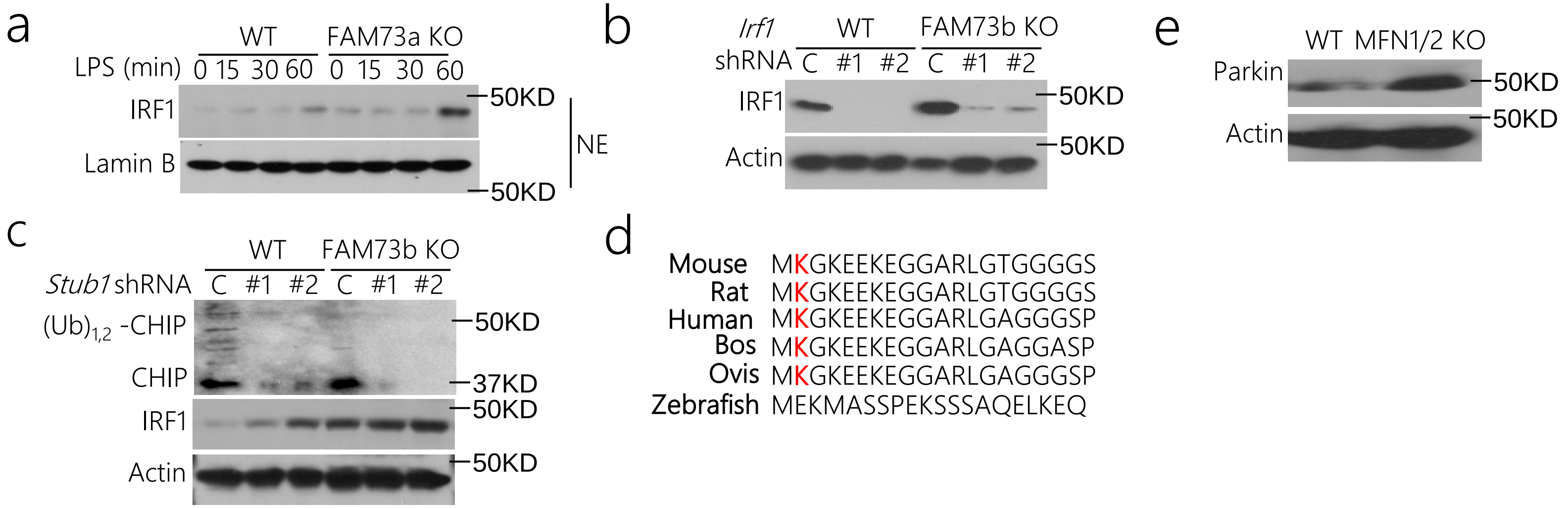

Supplementary Figure 8 Both of FAM73a *or* FAM73b control stability of CHIP-IRF1 (**a**) IB analyses of the indicated proteins nuclear (NE) extracts of WT and FAM73a KO BMDMs. (**b**) IRF1 level was analyzed by IB after infected with *Irf1*-specific shRNAs. (**c**) IB analysis of the CHIP and monoubiquitinated-CHIP in cytoplasmic (CE) and nuclear (NE) extracts of WT and FAM73b KO BMDMs stimulated with LPS. (**d**) Sequence alignment of identified K2 monoubiquitination site in CHIP orthologs of different species.(**e**) IB analyses of Parkin WT or MFN1/2 KO MEFs. Data are representative of three independent experiments.

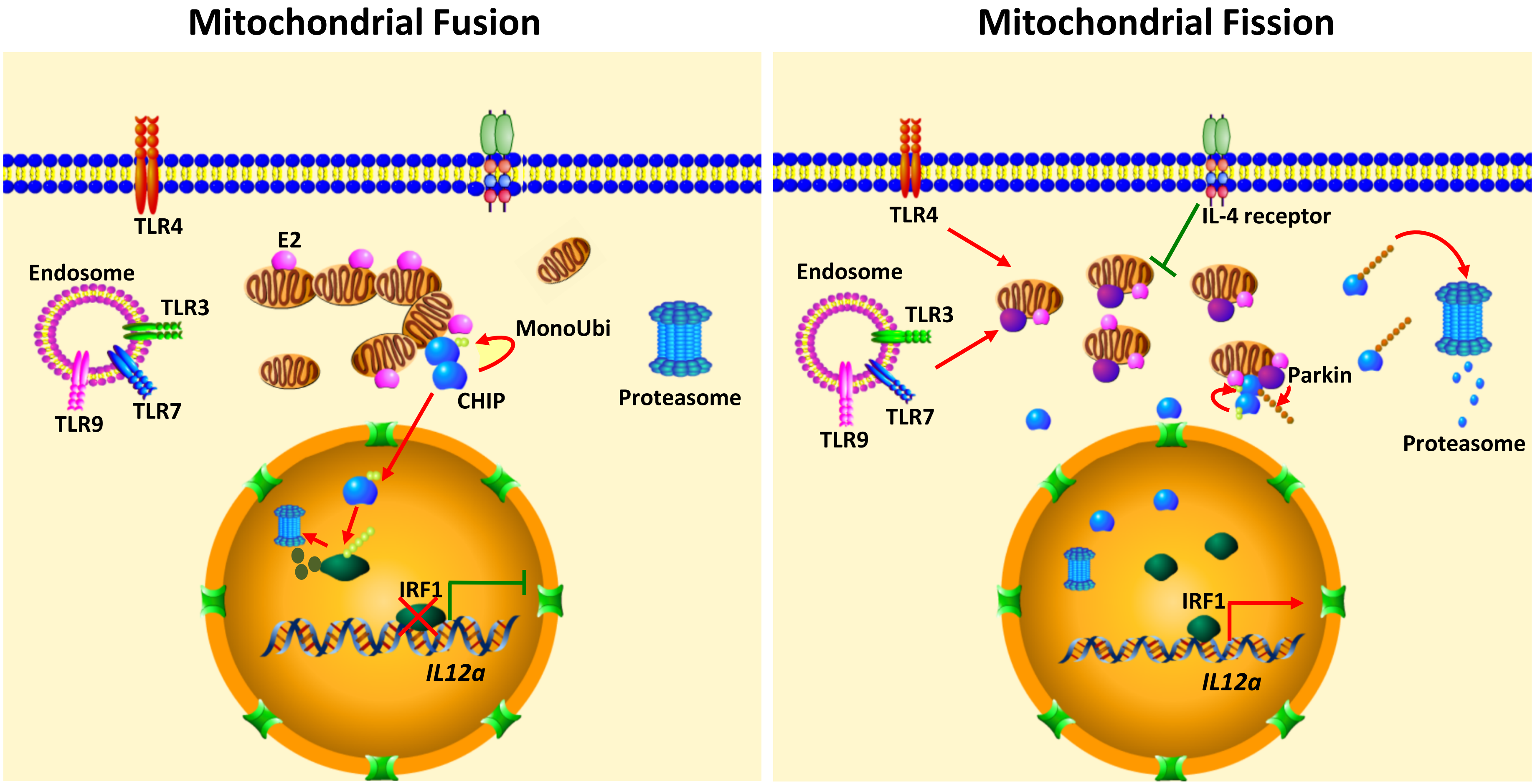

Supplementary Figure 9 A model of mitochondrial fission function in TLRs-mediated *Il12a* induction In fusion stage, monoubiquitinbated CHIP translocates to nucleus and induces IRF1 K48-linked polyubiquitination. Degradation of IRF1 specifically suppresses *Il12a* induction. Contrarily to resting cells, TLRs agonists induces the Mitochondrial fission rapidly in activated innate immune cells. Mitochondria fragments recruit Parkin, which promotes colocalized monoubiquitinated CHIP K48-linked ubiquitination and degradation. Released IRF1 binds to *Il12a* promoter and enhances its transcription. Similar mechanisms may apply to the induction of *Il10* and *Il23a* genes.

Fig.1e

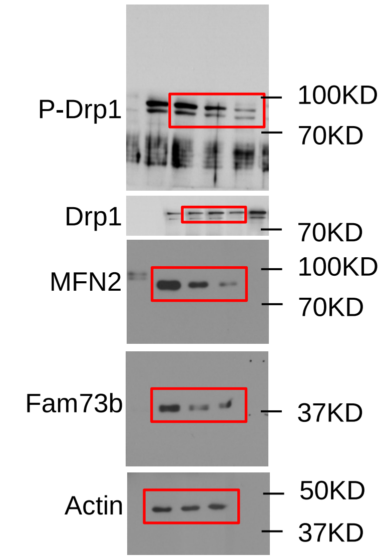

Fig.5a

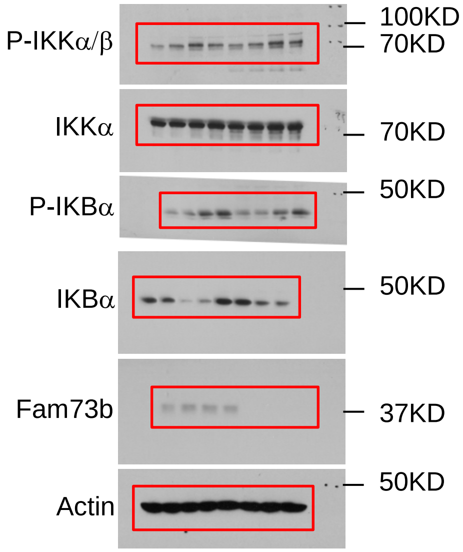

Fig.5b

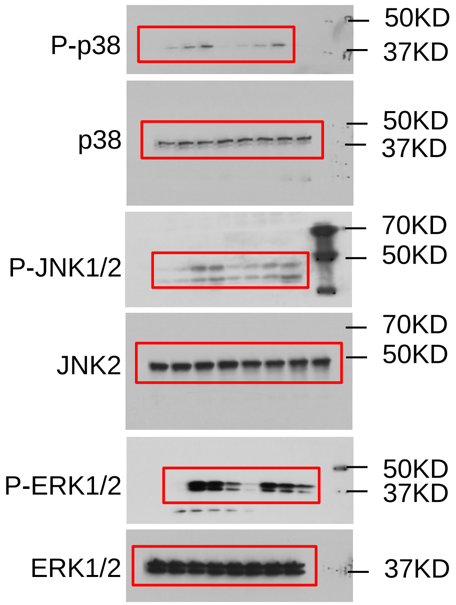

Fig.5c

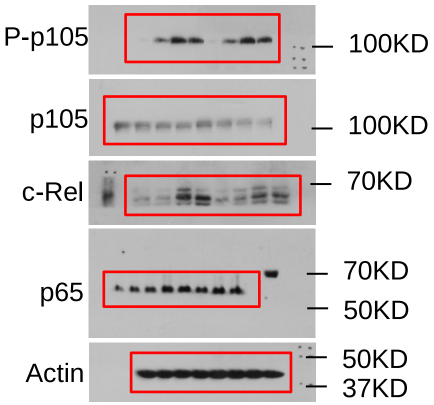

Fig.5c

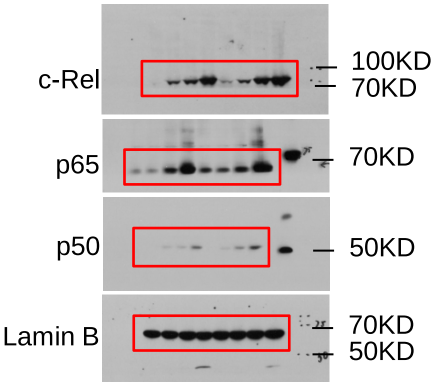

Fig.5d

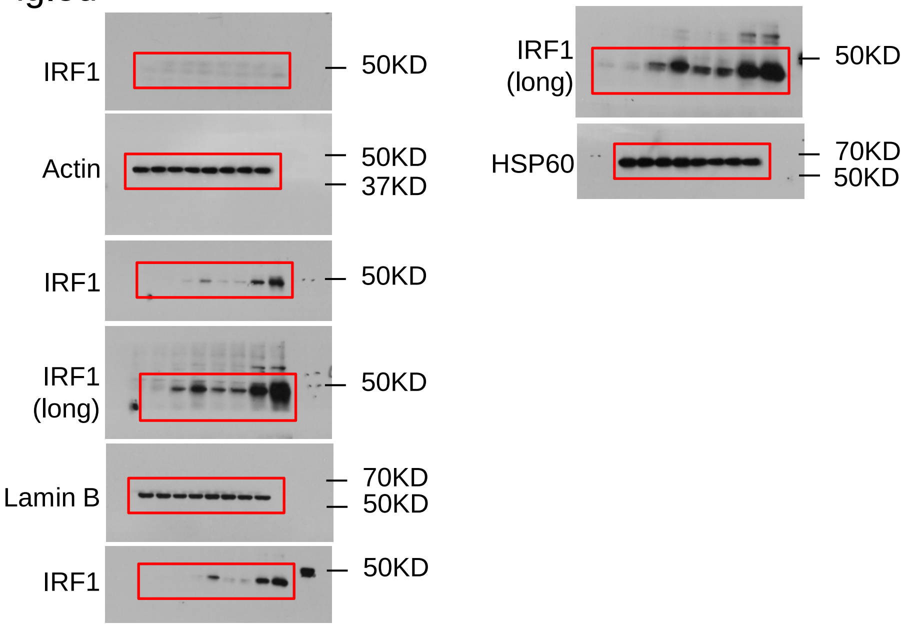

Fig.5f

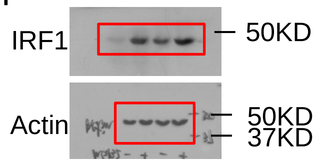

Fig.5i

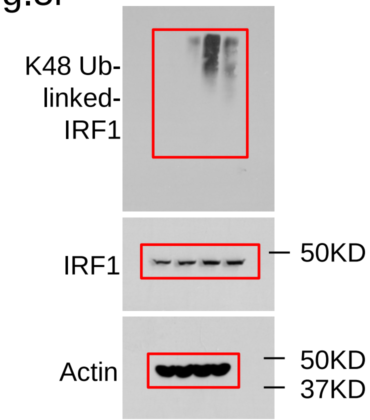

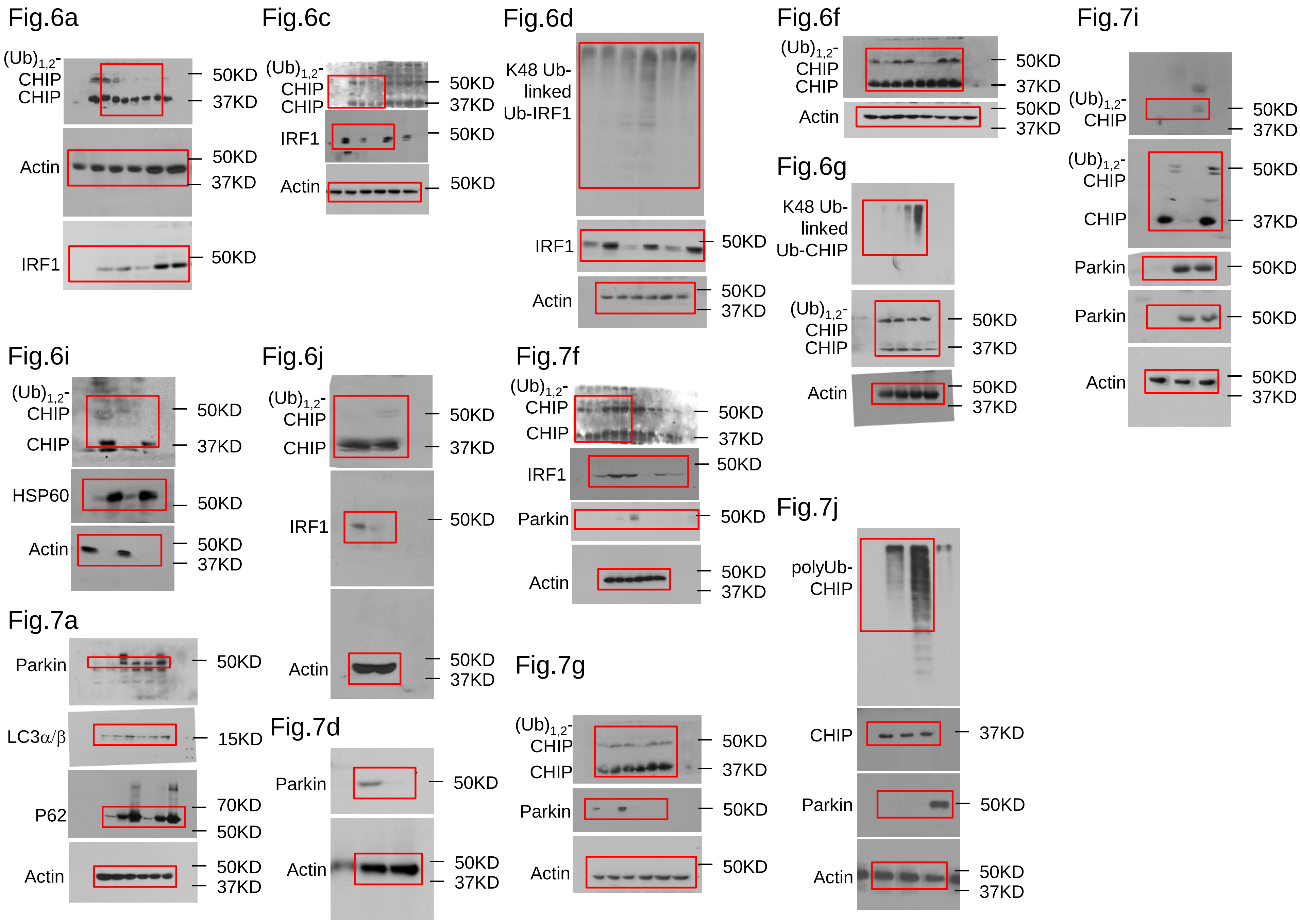
Fig.6a
(Ub)1,2-CHIP
CHIP
50KD
37KD
Actin
50KD
37KD
IRF1
50KD
Fig.6c
(Ub)1,2-CHIP
CHIP
50KD
37KD
IRF1
50KD
Actin
50KD
Fig.6d
K48 Ub-linked Ub-IRF1
IRF1
50KD
Actin
50KD
37KD
Fig.6f
(Ub)1,2-CHIP
CHIP
50KD
37KD
Actin
50KD
37KD
Fig.6g
K48 Ub-linked Ub-CHIP
(Ub)1,2-CHIP
CHIP
50KD
37KD
Actin
50KD
37KD
Fig.7i
(Ub)1,2-CHIP
50KD
37KD
(Ub)1,2-CHIP
CHIP
50KD
37KD
Parkin
50KD
Parkin
50KD
Actin
50KD
37KD
Fig.6i
(Ub)1,2-CHIP
CHIP
50KD
37KD
HSP60
50KD
Actin
50KD
37KD
Fig.6j
(Ub)1,2-CHIP
CHIP
50KD
37KD
IRF1
50KD
Actin
50KD
37KD
Fig.7f
(Ub)1,2-CHIP
CHIP
50KD
37KD
IRF1
50KD
Parkin
50KD
Actin
50KD
37KD
Fig.7j
polyUb-CHIP
CHIP
37KD
Parkin
50KD
Actin
50KD
37KD
Fig.7a
Parkin
50KD
LC3α/β
15KD
P62
70KD
50KD
Actin
50KD
37KD
Fig.7d
Parkin
50KD
Actin
50KD
37KD
Fig.7g
(Ub)1,2-CHIP
CHIP
50KD
37KD
Parkin
50KD
Actin
50KD

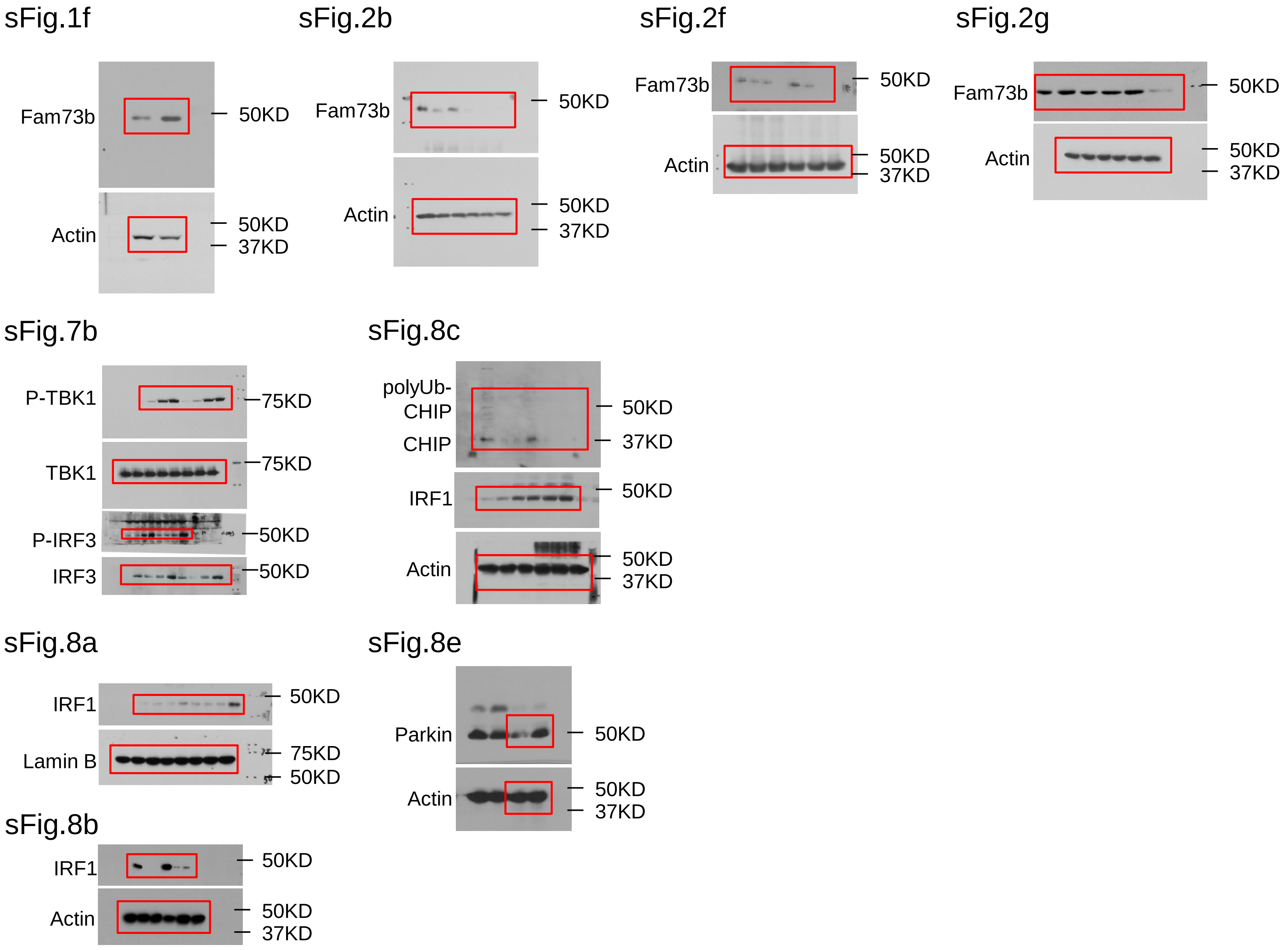

Supplementary Figure 10 All the uncropped scans of the western blots

Fig. 2h

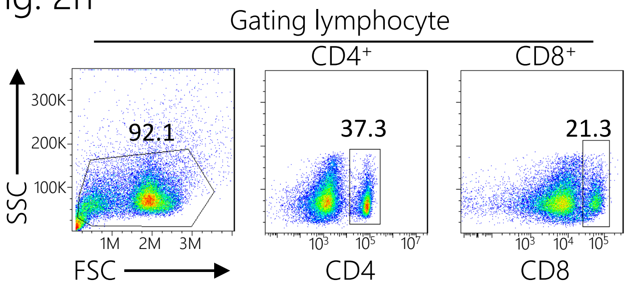

Fig. 3b

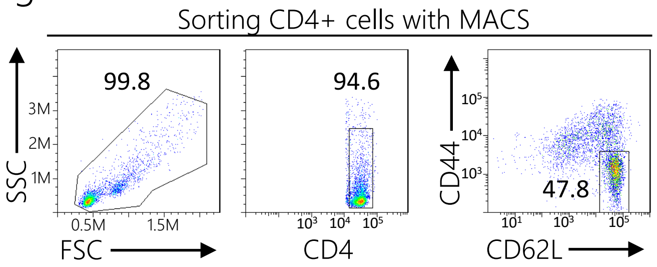

Fig. 3g-h

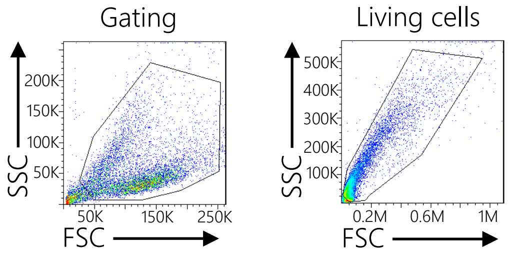

Fig. 3i

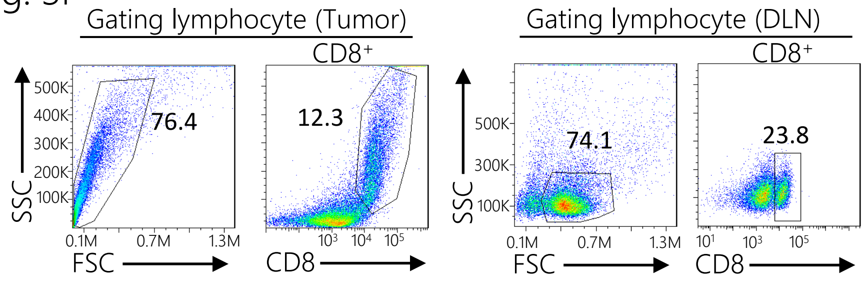

sFig.3a

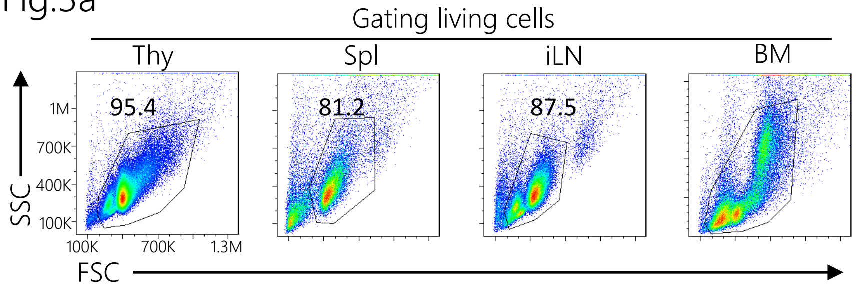

Supplementary Figure 11 All the FACS gating/sorting strategies

Supplementary Table 1 Gene-specific primers used in qRT-PCR experiments

Gene	Forward primer	Reverse primer
Il6	CACAGAGGATACCACTCCCAACA	TCCACGATTTCCCAGAGAACA
Tnf	CATCTTCTCAAAATTCGAGTGACAA	CCAGCTGCTCCTCCACTTG
Il12p35	ACTAGAGAGACTTCTTCCACAACAAGAG	GCACAGGGTCATCATCAAAGAC
Il12p40	GGAGACACCAGCAAAACGAT	TCCAGATTCAGACTCCAGGG
Il23p19	GCCAAGAAGAC CATTCCCGA	TCAGTGCTACAATCTTCTTCAGAGGACA
Il10	CCAGAGCCACATGCTCCTAGA	GGTCCTTTGTTTGAAAGAAAGTCTTC
Drp1	CGGTTCCCTAAACTTCACGA	GCACCATTTCATTTGTCACG
Fis1	CTACAGGGGTGCAGGAGAAA	AGATGGACTGGTAGGCATGG
Mfn1	CAGAGAAGAGGGTTTATTCA	ACTCATCAACCAAAACAGAT
Mfn2	TGAATGTTGTGTTCTTTCTG	AAGTGCTCTCTGCTAAATGT
Fam73a	GGGGACCACACCGCTCGGA	ACACGGGGAGTTCCAGGCCA
Fam73b	AGCCTCTATGTGCAAGGCAT	ACTCCCTTCGTTGGGACTCT
Irf1	CAGAGGAAAGAGAGAAAGTCC	CACACGGTGACAGTGCTGG
Arg1	TTTTTCCAGCAGACCAGCTT	AGAGATTATCGGAGCGCCTT
Ym1	TTTCTCCAGTGTAGCCATCCTT	TCTGGGTACAAGATCCCTGAA
Mrc1	CAGGTGTGGGCTCAGGTAGT	TGGCATGTCCTGGAATGAT
Fizz	TCCCAGTGCATATGGATGAGACCATA	CCTCTTCACTCGAGGGACAGTTGGCA
