# Supplementary material for: Mitochondrial dynamics controls anti-tumour innate immunity by regulating CHIP-IRF1 axis stability
Source: Nat Commun. 2017 Nov 27;8:1805. doi: 10.1038/s41467-017-01919-0 (PMC5703766; doi:10.1038/s41467-017-01919-0)
Supplement: Supplementary file 3 — Description of Additional Supplementary Files [file 41467_2017_1919_MOESM3_ESM.pdf]

**Description of Additional Supplementary Files**

File Name: Supplementary Data 1
Description: We isolated CD4+ Naïve T cells (CD4+ CD44- CD62Lhigh) from the spleens of WT and Fam73bfl/fl-CD4cre mice. The Gene expression was performed by RNAseq analysis.

File Name: Supplementary Data 2
Description: We isolated total bone marrow cells from WT and Fam73b-KO mice. The bone marrow-derived macrophages (BMDMs) were generated by M-CSF. After 5 days, BMDMs were starved for 12 hours and treated with LPS for other 6 hours. The Gene expression of nontreatment or LPS-induced groups was performed by RNAseq analysis.
